# Celecoxib increases EGF signaling in colon tumor associated fibroblasts, modulating EGFR expression and degradation

**DOI:** 10.18632/oncotarget.3678

**Published:** 2015-03-29

**Authors:** Roberta Venè, Francesca Tosetti, Simona Minghelli, Alessandro Poggi, Nicoletta Ferrari, Roberto Benelli

**Affiliations:** ^1^ Immunology Lab, IRCCS AOU San Martino - IST, Genoa, Italy; ^2^ Molecular Oncology and Angiogenesis Lab, IRCCS AOU San Martino - IST, Genoa, Italy

**Keywords:** EGFR, celecoxib, colon, fibroblast

## Abstract

We previously demonstrated that non-toxic doses of Celecoxib induced the immediate phosphorylation of Erk1-2 in colon tumor associated fibroblasts (TAFs), increasing their responsiveness to epidermal growth factor (EGF). We have now identified two concomitant mechanisms explaining the EGF-Celecoxib cooperation. We found that a 24-48h Celecoxib priming increased EGF receptor (EGFR) mRNA and protein levels in colon TAFs, promoting EGF binding and internalization. Celecoxib-primed TAFs showed a reduced EGFR degradation after EGF challenge. This delay corresponded to a deferred dissociation of EEA1 from EGFR positive endosomes and the accumulation of Rab7, pro Cathepsin-D and SQSTM1/p62, suggesting a shared bottleneck in the pathways of late-endosomes/autophagosomes maturation. Celecoxib modulated the levels of target proteins similarly to the inhibitors of endosome/lysosome acidification Bafilomycin-A1 and NH_4_Cl. Cytoplasmic vesicles fractionation showed a reduced maturation of Cathepsin-D in late endosomes and an increased content of EGFR and Rab7 in lysosomes of Celecoxib-treated TAFs.

Our data indicate a double mechanism mediating the increased response to EGF of colon TAFs treated with Celecoxib. While EGFR overexpression could be targeted using anti EGFR drugs, the effects on endosome trafficking and protein turnover represents a more elusive target and should be taken into account for any long-term therapy with Celecoxib.

## INTRODUCTION

Colon cancer is a slow developing tumor progressively altering tissue architecture. At all stages of tumor progression, colon TAFs almost invariably accompany and envelope epithelial cells, lining tubular crypts in well differentiated, early tumors or surrounding adenomatous crypts in advanced tumors [1-3]. The invasive front of advanced adenocarcinomas is also enriched in TAFs, taking an active part to tissue invasion as main producers of matrix metalloproteinases [4, 5]. The presence of an abundant TAF infiltration, at the invasive front of colon tumors, also predicts a reduced patient survival [6]. According to these observations, colon TAFs are not only an active and potentially malignant component of the tumor mass, but could be the first target of any drug, coming from the blood stream or the cell interstice. Anticancer drugs would challenge TAFs before reaching the neoplastic cell and TAFs could react to the drug, thus altering the microenvironment to limit epithelial damage [7, 8]. For this reason TAFs should be evaluated as drug targets.

Human colon is the body district more exposed to non-self antigens, due to the huge colonization by microbiota [9]. Alterations of gut microbiome can cause acute inflammatory responses and participate to chronic pathologies like inflammatory bowel disease, ulcerative colitis and colon cancer [10]. An abundant literature indicates inflammation as one of the leading causes of colon cancer and numerous clinical trials have shown that anti-inflammatory drugs effectively reduce colon tumor incidence [11]. Prostaglandin-endoperoxide synthases/cyclooxygenases (PTGS/COX) are the rate-limiting enzymes in prostaglandin (PG) and thromboxane neosynthesis [12]. While COX-1 is constitutively expressed in several organs, with an auto-limited biological activity (self-inactivation during the reduction of PGG2 to PGH2 [13]), COX-2 can be rapidly induced by mitogens and inflammatory stimuli [14]. COX-2 is frequently upregulated in colon cancer and is considered a prototypical target for chemoprevention [15]. Among specific COX-2 inhibitors, Celecoxib showed a powerful chemopreventive activity both against familiar and sporadic colon tumors [16, 17], though its long-term administration caused paradoxical effects both in mouse models [18, 19], activating gut fibroblasts, and in patients discontinuing the treatment [20], increasing adenoma occurrence.

EGFR is the prototype of the erb-B receptors family and the only one necessary for the correct development of the gut in transgenic knock-out mice [21, 22]. Many epithelial tumors rely on the activation of EGFR and in colorectal cancer EGFR gene amplification predicts a better response to the anti EGFR antibody therapy with Cetuximab [23]. EGFR levels are modulated not only by gene transcription, but also by its ligands. HB-EGF, Betacellulin and EGF induce a powerful short-term signaling, targeting EGFR for lysosomal degradation, while TGF-alpha, Epiregulin and Amphiregulin trigger a minor, but constant signaling by receptor recycling [24]. The activation and trafficking of EGFR, upon EGF binding, has been deeply investigated. EGF does not directly trigger EGFR dimerization, as observed for other growth factors, but induces a conformational change of the single receptor favoring dimerization. Both homo and heterodimers with other erb-B receptors can be formed. Dimerization induces the phosphorylation of multiple aminoacidic residues at the cytoplasmic carboxyl tail, activating several signaling pathways [22, 25]. Soon after activation, EGFR dimers are transferred to the early endosomes, that aggregate into larger vesicles by the concerted activity of the Early Endosome Antigen 1 (EEA1) and Ras-related GTP binding protein 5 (Rab5) [26]. Large endosomes mature by loosing Rab5 and EEA1, and acquiring Rab7, the GTPase that drives endosome-lysosome fusion [27, 28]. Of note, EGFR signaling is active unless large multivesicular bodies/late endosomes are formed and eventually fused with lysosomes [29, 30]. Targeting EGFR to degradation without activation is the therapeutic goal of Cetuximab, being the disturbance of internalization/degradation a major mechanism for acquired resistance to this drug [31].

Previously, we characterized the effects of Celecoxib on human colon TAFs showing that nontoxic doses (up to 25μM) were able to increase colon TAFs proliferation in the presence of EGF [32]. Celecoxib sustained EGF signaling without affecting EGFR phosphorylation, but increasing Erk1-2 activity. EGFR or Erk1-2 inhibitors were able to inhibit the Celecoxib + EGF synergy, thus blocking TAFs proliferation. We also found that the kinetic of Erk1-2 activation by Celecoxib was bimodal with a strong and transient early response, followed after 24h, by a lower and constitutive reactivation. These findings suggested two distinct mechanisms mediating the synergy of Celecoxib with EGF: the first one direct and the other mediated by gene expression. Here we further investigated the synergy between Celecoxib and EGF-mediated signaling in TAFs, focusing on EGFR. Celecoxib increased total EGFR levels in colon TAFs by both inducing EGFR neosynthesis and by slowing down EGF-triggered EGFR degradation. The latter effect was apparently mediated by a retarded maturation of the late endo-lysosomal degradative compartment. As a consequence of this altered vesicle maturation, Celecoxib could modulate not only the amount of EGFR, but also the intracellular levels of other proteins degraded through the same pathway. This might alter the functional behavior of colon TAFs and trigger unpredictable responses to external stimuli such as the drugs used in colon cancer therapy.

## RESULTS

### Celecoxib activates colon TAFs and increases EGF-triggered responses

**Figure 1:**
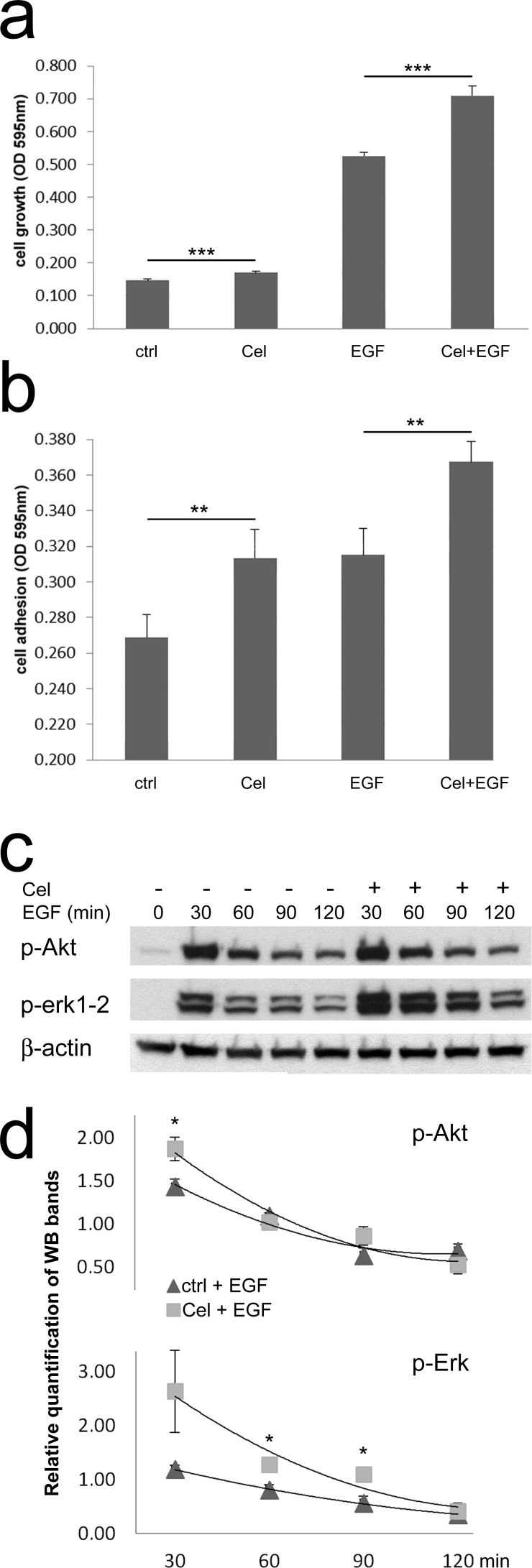
Celecoxib increases colon TAFs responsiveness to EGF **a**) TAF cell growth was evaluated on day 7 of culture in the presence of Celecoxib (Cel, 10μM), EGF (50ng/ml) or Cel+EGF. Ctrl: control TAFs in absence of stimuli. The test was run in six replicates and repeated three times. **b**) Cell adhesion of TAFs seeded on collagen type IV. TAFs were primed or not with Celecoxib in culture flasks, afterwards they were plated in microwells either without additional treatment or with EGF, for 30min. The test was run in quadruplicates and repeated three times. **c**) Western blot for p-Akt and p-Erk1-2 of TAFs primed with Celecoxib and/or incubated with EGF for the indicated period of time. Beta-actin was used as a loading control. **d**) Relative quantification of WB replicates run as shown in panel c.

We previously showed that colon TAFs exhibit a great tolerance to Celecoxib treatment. Moreover, Celecoxib at nontoxic concentration activated colon TAFs signaling, causing a rapid and transient phosphorylation of Erk1-2 [32]. This activation was able to synergize with low dose EGF (1ng/ml). In this study we first assessed whether Celecoxib could affect TAFs growth also in the presence of the high dose EGF, used here (Fig. 1a). Celecoxib induced a negligible effect *per se*, but it consistently increased the EGF-mediated triggering of TAFs growth. To analyze whether Celecoxib could affect TAFs adhesive properties, a feature of TAFs activation, we assessed binding of TAFs to collagen (Fig. 1b). Of note, Celecoxib alone increased TAFs adhesion to collagen as compared to untreated cells. More importantly, Celecoxib potentiated EGF-mediated adhesion to collagen. This effect was associated with a more intense activation of Erk1-2 phosphorylation induced by Celecoxib and EGF together, than that induced by EGF alone. On the other hand, Akt phosphorylation consequent to EGF signaling was poorly affected by Celecoxib (Fig. 1c and 1d).

### Celecoxib increases EGFR mRNA and protein expression in colon TAFs

**Figure 2:**
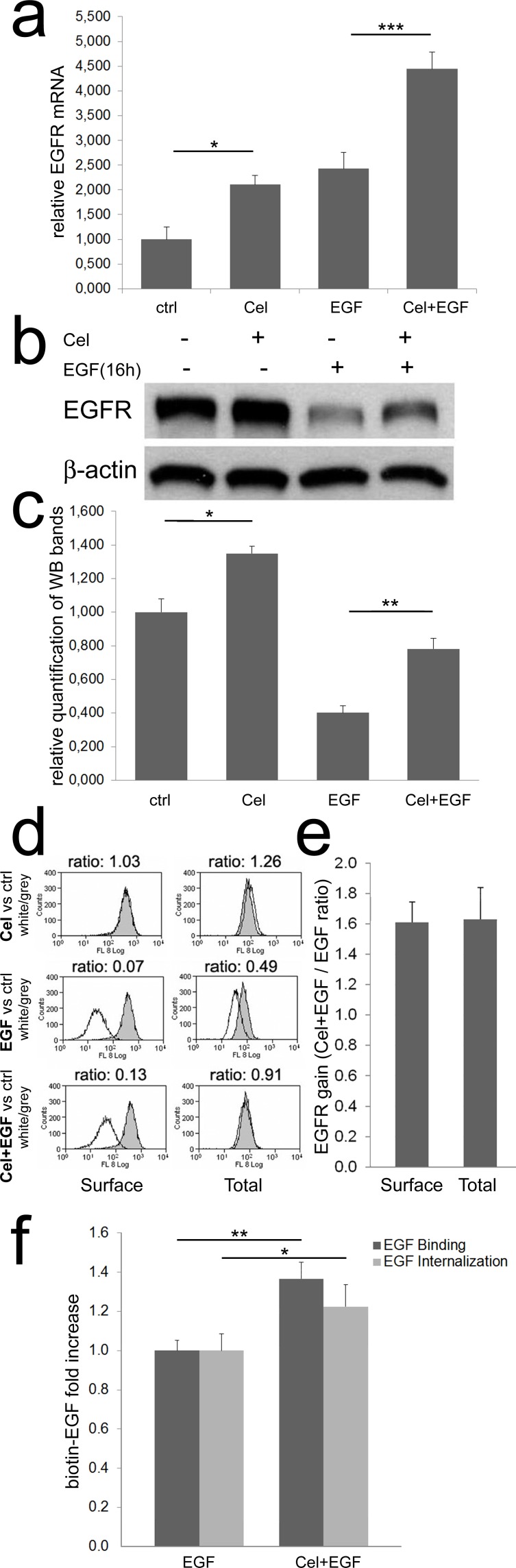
Celecoxib increases EGFR mRNA and protein expression **a**) Real time PCR for EGFR. Colon TAFs were treated with Celecoxib (Cel, 10μM) for 48 hours; EGF (50ng/ml) was added as indicated during the last 16h of incubation. EGFR mRNA levels were normalized against the RP2 housekeeping gene. The mean values of three independent tests are shown. **b**) A western blot for EGFR under the same condition reported for Real Time PCR. **c**) The relative mean intensity of bands from six independent western blots, on three different TAFs primary cell cultures, was calculated by densitometry and plotted. **d**) Flow cytometric analysis of surface and total EGFR. TAFs were treated as described above. The peaks, representing EGFR expression under Celecoxib (Cel), EGF, or Celecoxib plus EGF (Cel+EGF) treatments (white peaks), were compared to EGFR levels detected in untreated controls (grey peaks). The MFI ratio (white/grey) was calculated and reported on each panel. **e**) Surface and total EGFR increase induced by Celecoxib, calculated as Cel+EGF / EGF ratio of three independent flow cytometric analyses as reported in panel d. **f**) Binding and internalization of biotin-EGF in colon TAFs primed or not with Celecoxib and treated with biotin-EGF (50ng/ml) for 45 or 30min respectively. The test was run in six replicates and repeated three times.

Previously, we observed that long-term pretreatment of colon TAFs with Celecoxib apparently caused EGFR upregulation. To define whether Celecoxib could modify EGFR mRNA levels, Real Time PCR analysis was performed. As shown in Fig. 2a, EGF increased the expression of its receptor in colon TAFs, and Celecoxib approximately doubled EGFR mRNA both in untreated, or EGF-treated TAFs. At protein level (Fig. 2b and 2c), EGF caused a massive degradation of its receptor after an overnight incubation. Celecoxib increased EGFR levels either in controls and, with higher efficiency, in EGF-treated TAFs. The EGFR-inducing activity of Celecoxib was not confined to colon TAFs, as also normal fibroblasts from colon mucosa and some CRC cell lines showed similar results, with some exceptions (supplementary Fig. 1S).

To confirm western blot findings, colon TAFs were analyzed in the same experimental settings by flow cytometry, for quantification of surface-expressed and total EGFR. Under chronic Celecoxib regimen alone, a modest increase of total EGFR was detectable by this method (Fig. 2d, upper row of histograms). EGF chronic treatment induced internalization and degradation of EGFR with the consequent decrease of both surface and total EGFR levels (Fig. 2d, middle row). In the presence of EGF, Celecoxib caused a strong rescue of EGFR, clearly evident for total EGFR, and proportionally identical for the surface-bound EGFR fraction (Fig. 2d, lower row). The mean of three distinct experiments on two different TAFs primary cell cultures showed an almost constant 1,6 fold increase of EGFR in the presence of Celecoxib under EGF chronic treatment, both as surface- expressed or total protein level (Fig. 2e). Celecoxib-triggered increase of EGFR protein levels, found under EGF treatment, was higher as compared to the effect of Celecoxib alone in controls. This gain could be driven by an impaired interaction of EGF with its receptor, or by a limited internalization of their complex. For this reason we performed binding and internalization assays using biotinylated EGF. We found that Celecoxib-induced increase of EGFR protein was coupled to an improved ability of colon TAFs to bind and internalize EGF, excluding any direct influence of Celecoxib on EGF-EGFR interaction (Fig. 2f).

### Celecoxib delays EGFR degradation

The increase of EGFR induced by Celecoxib was more evident in the presence of an elevated turnover triggered by EGF, suggesting the coexistence of different mechanisms modulating EGFR kinetic other than the induction of EGFR mRNA transcript.

**Figure 3:**
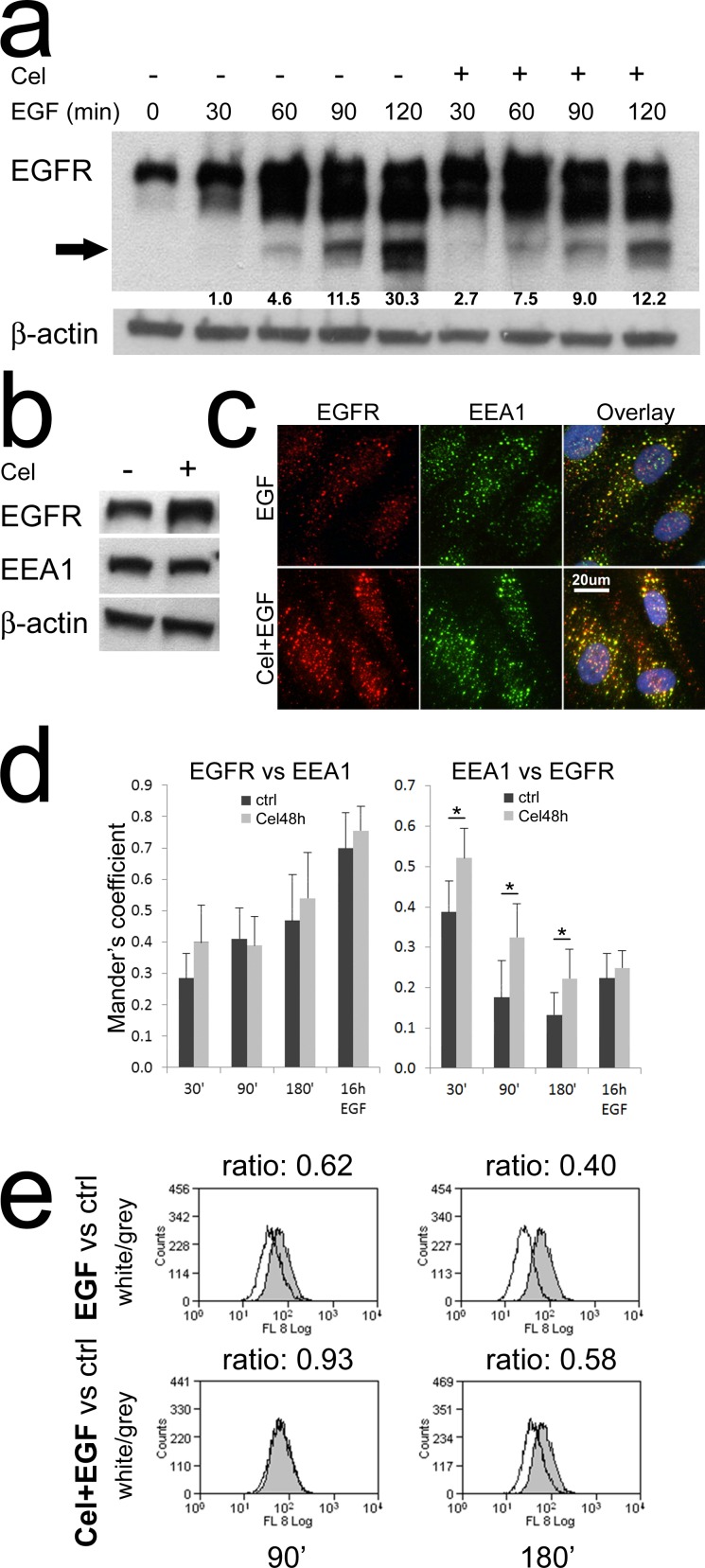
Celecoxib slows down EGFR degradation **a**) Western blot analysis of the kinetic of EGFR degradation. Colon TAFs pretreated with Celecoxib were challenged with EGF for the indicated times. The arrow indicates the band used for EGFR degradation quantification. The test was repeated twice. **b**) The early endosome marker 1 (EEA1) levels were not influenced by Celecoxib pretreatment. **c**) A representative image (90min EGF) of double immunofluorescence analyses: EGFR (red), EEA1 (green). Celecoxib-pretreated colon TAFs were challenged with EGF for 30, 90, 180min or 16h. Fluorescent images were acquired, with fixed expositions (EEA1-488 f1/8; EGFR-594 f1/3; DAPI f1/100), by a Leica DM-LB2 microscope equipped with I3 and M2 filters and a HCX PL Fluotar 40x non immersion optic. A 20μm scale is shown. **d**) Analysis of Mander's overlay coefficients for EGFR and EEA1 on the double immunofluorescence. Six random 40x fields per condition -containing at least 12 TAFs- were analyzed (see methods). The test was repeated twice. **e**) Flow cytometric analysis for EGFR expression in TAFs pretreated or not with Celecoxib and then challenged for 90 or 180min with EGF. The peaks, representing EGFR expression under EGF, or Celecoxib plus EGF (Cel+EGF) treatments (white peaks), were compared to EGFR levels detected in untreated controls (grey peaks). The MFI ratio (white/grey) was calculated and reported on each panel. The test was repeated twice.

We tested if Celecoxib could alter the kinetic of EGFR degradation upon EGF triggering. Challenging colon TAFs with EGF (Fig. 3a), we noticed the appearance of low molecular weight bands indicating EGFR degradation at 60min and 90min, becoming more evident at 120min. Interestingly, in the cells pretreated with Celecoxib EGFR degradation was increased during the first hour of triggering, but delayed at 90 and even more at 120min. To track EGFR along its degradative route we needed an endosomal marker whose levels were not modulated by Celecoxib. We found that the early endosome marker 1 (EEA1) satisfied this condition (Fig. 3b), thus EEA1 was used for co-localization studies with EGFR. By double immunofluorescence and microscopic evaluation, we analyzed the overlap for EGFR and EEA1 signals in colon TAFs. The representative images shown in Fig. 3c (90min of incubation with EGF) indicated that most EGFR staining localized in EEA1 positive vesicles. Calculating Mander's coefficients for EEA1 and EGFR co-localization (Fig. 3d) it was evident that EGFR co-localization with EEA1 increased over time and it was unaffected by Celecoxib (left panel). This observation confirmed the unaltered internalization of the receptor upon EGF binding. On the contrary EEA1 dissociated from EGFR less efficiently in the presence of Celecoxib (right panel).

Flow cytometry analyses of total EGFR levels in the same experimental conditions (Fig. 3e), showed that the delayed dissociation of EEA1 from EGFR-positive endosomes was associated with a reduced degradation of EGFR in Celecoxib-treated samples as compared to controls, despite a progressive loss of the receptor over time in both conditions.

### Celecoxib alters protein turnover

**Figure 4:**
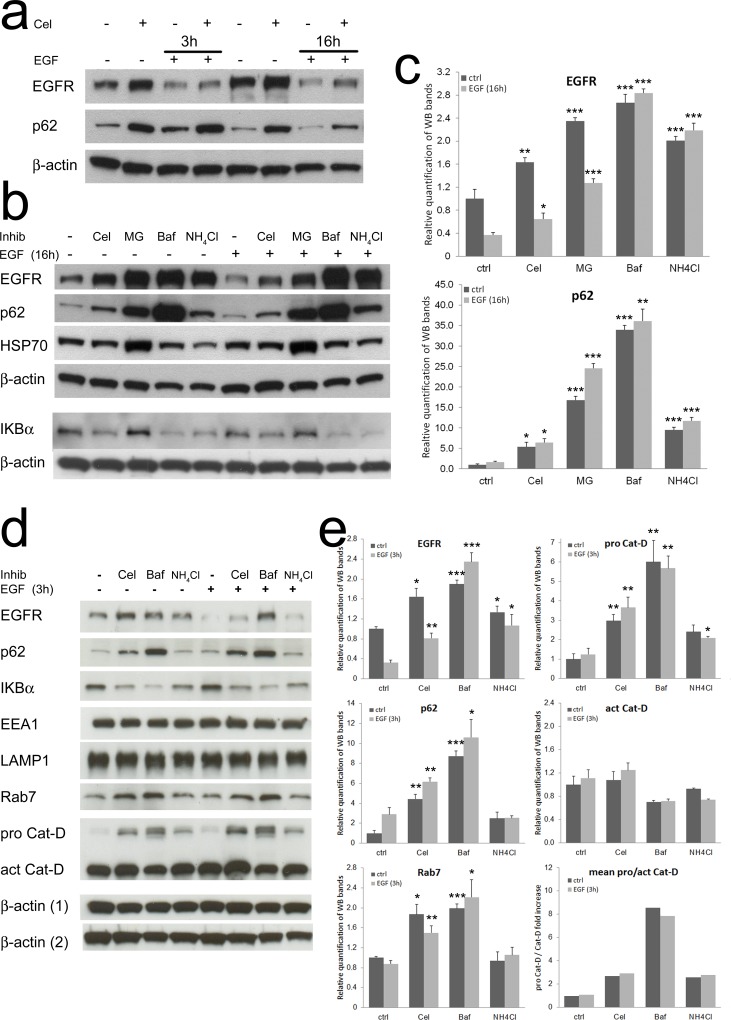
Celecoxib affects protein turnover mimicking the inhibitors of endo-lysosome acidification **a**) Western blot for EGFR and p62/SQSTM1 of colon TAFs pretreated with Celecoxib and triggered for 3 or 16h with EGF. **b**) Western blot analysis of the effects of proteasome-lysosome inhibitors. 48h treatment with MG132 (proteasome inhibitor, 2μM), Bafilomycin-A1 (Baf, V-ATPase inhibitor, 25nM), NH_4_Cl (lysosomal pH -buffering molecule, 10mM) were compared to Celecoxib (10μM) as modulators of EGFR, p62, HSP70 and IkBα. **c**) Relative quantification of EGFR and p62 levels from replicate tests as shown in panel b; p values were calculated as compared to untreated controls. **d**) Western blot comparison of the effects of Celecoxib and lysosome acidification inhibitors. The effects of Celecoxib (10μM), Bafilomycin-A1 (2.5nM) and NH_4_Cl (2.5mM) pretreatment were tested in the absence/presence of EGF (3h) on several target proteins: EGFR, p62, IkBα, EEA1, LAMP1, Rab7, pro Cathepsin-D and Cathepsin-D. Loading controls: beta-actin (1) normalizes EEA1, p62 and Cathepsin-D; beta-actin (2) normalizes EGFR, IkBα, LAMP1 and Rab7. **e**) Relative quantification of EGFR, p62, Rab7, pro and active Cathepsin-D levels from replicate tests as shown in panel d; p values defined in Materials and Methods were calculated as compared to untreated controls.

The main cellular routes for protein degradation are the proteasome [33], a protein complex directly acting on cytoplasmic proteins, and the lysosome [34], a vesicle-delimited compartment collecting both extracellular and membrane bound proteins (by endosomes), and cytoplasmic proteins (by autophagosomes). These pathways are partially interdependent and cooperate to determine the correct turnover of several proteins. EGF-induced EGFR degradation is both proteasome- and lysosome-dependent [35-37]. Thus, we looked for a marker protein modulated by both proteasome and lysosome activity, suitable as a shared sensor for these pathways. p62/SQSTM1 is an ubiquitin binding protein triggering protein and organelles degradation through autophagy [38]. P62 is incremented upon proteasome or lysosome inhibition [39, 40], we then decided to evaluate its levels in our experimental system. Western blot analysis (Fig. 4a) showed a striking accumulation of p62 protein in the presence of Celecoxib. P62 was poorly affected by EGF triggering. Double immunofluorescence of p62 and EGFR showed a low number of active autophagosomes per cell (Fig. 2S a), with very rare single cells showing high number of p62 positive vesicles. P62 and EGFR showed a reduced co-localization in colon TAFs primed with EGF even in the presence of Celecoxib (Fig. 2S b). This observation indicated a marginal participation of p62 and autophagy to EGFR degradation in our model. Accordingly, we could hypothesize that EGFR and p62 were independently subdued to the same effect of Celecoxib on protein turnover.

To further investigate the activity of Celecoxib on protein degradation pathways, we compared Celecoxib with inhibitors targeting the proteasome (MG132), endosome acidification by inhibition of the vacuolar type H+/ATPase or V-ATPase (Bafilomycin-A1) and neutralization of lysosomal pH (NH_4_Cl). EGFR, p62, HSP70 and IkBα were used as protein targets (Fig. 4b). HSP70 and IkBα were selected as known targets for proteasome-mediated degradation [41, 42]. We observed that Celecoxib increased EGFR and p62 levels (quantified in Fig. 4c), leaving unaffected HSP70 and apparently lowering IkBα. MG132 increased all these marker proteins. Bafilomycin-A1 and NH_4_Cl displayed a similar behavior as compared to Celecoxib. Further, as expected, only EGFR was substantially lowered by EGF treatment. These findings suggested that Celecoxib could affect endosome maturation by influencing vesicular acidification. Thus, we quantified the pH variations induced by Celecoxib using the Lysosensor-Green DND-189 probe; unfortunately, Lysosensor-Green stained TAFs cytoplasm, giving an excessive background noise at any concentration tested, rendering inadequate this experimental approach (not shown).

We then decided to analyze markers of endocytic vesicles involved in protein turnover. In these experiments we lowered Bafilomycin-A1 and NH_4_Cl concentrations to obtain an increase of EGFR levels comparable to that achieved with Celecoxib. We also limited EGF triggering (3h) to maximize the effects on EGFR degradation, reducing the contribution of its neosynthesis (Fig. 4d). The inhibitors modulated EGFR and p62 levels coherently with the previous experiments (Fig. 4e). The analysis of endocellular vesicles markers showed stable EEA1 (early endosome) and LAMP1 (late endosome/lysosome) levels. On the contrary, the late endosome marker Rab7 was particularly increased in the presence of Celecoxib and Bafilomycin-A1, showing a modulation similar to EGFR (Fig. 4e, bottom-left panel). This modulation was suggestive of a delay in late endosomes maturation possibly causing their accumulation and a retarded fusion with lysosomes [27, 30], and accounting for impaired EGFR degradation. Rab7-enriched late endosomes tend to cluster favoring homotypic tethering that lowers the efficiency of movement and delivery of cargo molecules from multivesicular bodies (MVB) to lysosomes [43]. To further explore this hypothesis, we evaluated pro- and active Cathepsin-D levels. Cathepsin-D is an aspartic protease that accumulates in endosomes/phagosomes as an immature pro-peptide and is activated by acidic enzymes at low pH [44, 45], so it can be used as an indirect marker of endosome maturation and acidification. We found that Celecoxib, Bafilomycin-A1 and NH_4_Cl induced an EGF independent increase of pro-Cathepsin-D (Fig. 4e upper-right panel). While Bafilomycin-A1 caused an evident switch in favor of pro Cathepsin-D, lowering the processing to active Cathepsin-D (Fig. 4e middle and bottom-right panel), Celecoxib and NH_4_Cl apparently caused an increment of the pro-enzyme without lowering its active form.

### Celecoxib retards pro Cathepsin-D activation in late endosomes

**Figure 5:**
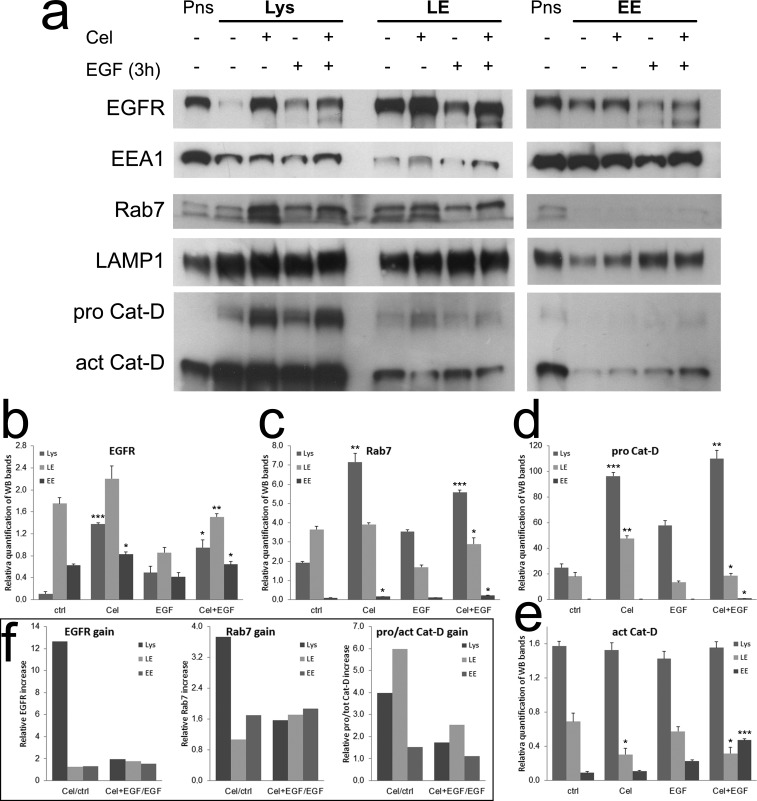
Celecoxib retards Cathepsin-D maturation in late endosomes, causing EGFR and Rab7 accumulation **a**) Cytoplasmic vesicles from TAFs treated as shown were fractioned by centrifugation (see methods) obtaining samples enriched in lysosomes (Lys: EEA1 low; Rab7, LAMP1 high; Cathepsin-D very high), late endosomes (LE: EEA1 low; Cathepsin middle; Rab7, LAMP1 high) and early endosomes (EE: EEA1 high; Rab7, LAMP1, Cathepsin-D low). Post nuclear supernatants (Pns) from untreated controls were used as loading controls. **b**-**e**) Relative quantification of EGFR, Rab7, pro Cathepsin-D and active Cathepsin-D bands in Lys, LE and EE-enriched fractions from replicates of the test shown in panel a. f) EGFR, Rab7 and pro/active Cathepsin-D gain in the Lys, LE and EE-enriched fractions of Celecoxib-treated samples, calculated from data shown in graphs b-e as ratios against the relative controls (both untreated or EGF-treated).

The data on Rab7 and Cathepsin-D obtained with drugs contrasting endo-lysosomal acidification, suggested that Celecoxib could retard EGFR degradation by the same mechanism, causing a delay in pro Cathepsin-D cleavage. As this was not evident in whole cell lysates, we purified fractions of cytoplasmic vesicles enriched in early endosomes (EE), late endosomes (LE) and lysosomes (Lys) to verify the levels of EGFR, Rab7 and pro/active Cathepsin-D in each compartment (Fig. 5a). In all experimental conditions the main quote of EGFR localized in LE (Fig. 5b), followed by Lys. In these compartments the increment induced by Celecoxib was more evident as compared to EE. Rab7 correctly localized in LE and Lys (Fig. 5c), Celecoxib was active on both compartments increasing Rab7 levels especially in the lysosome -enriched fraction, as compared to relative controls. When we analyzed the levels of pro and active Cathepsin-D (Fig. 5d and 5e), we found most of the enzyme correctly localized in Lys and LE-enriched fractions. In LE we observed that Celecoxib lowered the levels of the active enzyme as compared to controls, while pro Cathepsin-D was increased, confirming a block of its maturation. To summarize these observations, we calculated the ratios of Celecoxib-treated samples against the relative controls (both untreated or EGF-treated, Fig. 5f). This graphical representation made evident that, in the absence of EGF, the accumulation of immature Cathepsin-D in LE was accompanied by a robust increase of Rab7 and intact EGFR in the Lys-enriched fraction of Celecoxib-treated TAFs. The chronic presence of high dose EGF was able to unclog vesicle trafficking and EGFR degradation, though the retard in Cathepsin-D maturation in LE was maintained by Celecoxib, and both EGFR and Rab7 levels exceeded controls.

## DISCUSSION

Despite the advances in early diagnosis and therapy, colorectal cancer remains a big killer among solid tumors, with only 50% of patients reaching a 5-year survival after curative surgery. The therapeutic protocols based on 5-fluorouracil and platinum have not been significantly implemented during the last five years, while targeted therapy with biological agents is applied with high costs and marginal effects against the metastatic disease [11]. A possible way to circumvent these biases is chemoprevention. As sporadic colon cancer is a slow developing tumor of the elderly, chemoprevention could radically change its incidence and natural history, possibly delaying the occurrence of aggressive tumors to an age that exceeds natural death. A well-defined target of chemoprevention is COX-2, an enzyme induced by inflammation, hypoxia and stress signals. COX-2 catalyzes the rate-limiting step of prostaglandin E2 (PGE2) neo synthesis and has been extensively linked to colon cancer [46]. COX-2 expression regulation is extremely complex and its direct linkage to colon cancer aggressiveness in late stages is not definitely proven [47, 48], though several NSAIDS, such as aspirin and Celecoxib, have shown strong chemopreventive effects on colon tumors [16, 17, 49-51]. While the side effects of these drugs have temporary biased their extended use in chronic regimens, the recent finding of a correlation between PIK3CA mutation in colon tumors and an increased survival of patients under long-term aspirin therapy at diagnosis [52], indicates the need for specific markers identifying responders to NSAIDS regimen.

Celecoxib has been extensively tested *in vitro* on colon cancer cell lines, showing both COX-2 dependent and independent effects [53-55]. While these observations are useful in the context of advanced cancer models, they do not reflect the pathophysiology of normal mucosa and early adenomas, where COX-2 is mainly expressed in the stroma [56-60]. In the min^−/+^ mouse model continued long-term Celecoxib regimen caused an initial regression of intestinal tumors, but finally the incidence was comparable to untreated controls [19]. This failure of chemoprevention was accompanied by a strong activation of gut fibroblasts and tissue fibrosis [18, 61]. We thus decided to test Celecoxib on primary human colon TAFs, identifying a strong activation of Erk1-2 and a powerful synergy with EGF [32].

EGFR is deregulated in most epithelial tumors [62]. In colorectal cancer EGFR is rarely mutated, while gene amplification is more frequent and associates to a better response to anti EGFR monoclonal antibodies [23, 63, 64]. Both colon tumor epithelial cells and TAFs share EGFR expression. In our hands, colon TAFs were more responsive to EGF as compared to bFGF [32] suggesting that, in the presence of an anti EGFR therapy, they could be efficiently targeted. Indeed, we reported that both Cetuximab and the EGFR tyrosine kinase inhibitor Thyrphostin were able to inhibit the Celecoxib + EGF synergy. Despite the evident amplifying effect exerted by Celecoxib on EGF activity, we were unable to characterize a direct influence of Celecoxib on EGFR phosphorylation [32].

In the present study, we show that a long-term treatment with Celecoxib is able to increase the levels of total EGFR in colon TAFs. This increment could explain the synergy of Celecoxib with EGF that results particularly evident when colon TAFs are exposed to EGFR triggering. The gain in EGFR caused by Celecoxib under EGF treatment is not only mediated by an active transcription of the receptor, but it is also accompanied by a retarded degradation.

EGFR has been extensively studied as a prototype of growth factor receptor activation and trafficking [65]. EGFR, upon EGF binding, forms active dimers with multiple phosphorylated residues at the cytoplasmic carboxyl tail [25]. These residues act as docking stations that activate several signaling pathways. Phosphotyrosine 1045 in particular recruits cbl, triggering the ubiquitination of EGFR and its sorting to lysosomes for degradation [66]. EGFR can be internalized by both a clathrin-dependent or independent route. The former is usually activated by low concentrations of EGF and allows for receptor recycling, the latter is triggered by high EGF concentrations (our experimental condition) and drives EGFR to degradation [67, 68]. Endocytosed vesicles fuse to early endosomes where EGFR continues to signal by its carboxyl-terminal tale facing the cytoplasm. While the pH of endosomes is progressively lowered by V-ATPase, the receptor does not dissociate from EGF, due to the high affinity of their binding [24]. The signaling of EGFR is stopped only in the MVBs of the late endosomal compartment, where the receptor is separated from the cytoplasm [29]. Finally, the fusion of late endosomes with lysosomes mediated by the small GTPase Rab7, causes the complete degradation of EGFR and its ligand [30].

According to our results, Celecoxib can affect different steps of this pathway. The neo-synthetic increase of total EGFR can favor EGF binding and receptor activation, causing an initial empowerment of internalization and signaling (Fig. 1c, 1d, Fig. 2f and Fig. 3a at 30′). This early increased signal has been shown to cause a negative feedback, switching off EGFR signaling [69] and enhancing EGFR degradation [67], however this was not observed in our experimental model. On the contrary, the panels a and e of Fig. 3 show a retarded degradation of EGFR in the presence of Celecoxib. At the same time, the immunofluorescence analysis indicates a persistent co-localization of EEA1 with EGFR in the medium-large endosomes of Celecoxib-treated TAFs, as compared to controls. The delayed negativization of EEA1 in EGFR-positive endosomes suggests a lag in endosomes maturation, while the linear increase of EGFR co-localizing with EEA1 indicates that EGFR internalization is not negatively affected by Celecoxib pretreatment, as also confirmed by binding and internalization assays showing an increased activity of the receptor (Fig. 2f). The retarded degradation of EGFR is particularly evident between 90 and 180min from EGF triggering, suggesting that Celecoxib could influence the maturation in terms of pH acidification and progression toward lysosome fusion of late endosomes. This hypothesis is sustained by the enrichment of Rab7, pro Cathepsin-D and p62 in Celecoxib-pretreated samples. The accumulation of Rab7 positive endosomes suggests an impaired fusion with lysosomes [27], accompanied by pro Cathepsin-D rise, an enzyme typically activated by low pH [44, 45]. Autophagosomes share with endosomes the enrichment in Rab7 and Cathepsin-D during maturation [70, 71]. The increase of the sequestosome-1 protein p62, that participates to the formation of autophagosomes [39], suggests that the inhibitory activity of Celecoxib can be exerted at a late step of endosomes/autophagosomes maturation, sharing Rab7 and Cathepsin-D enrichment.

We also observed that the V-ATPase inhibitor Bafilomycin-A1 and the lysosome inhibitor NH_4_Cl mimic Celecoxib activity modulating the levels of several marker proteins. Further, using cytoplasmic vesicle fractionation and pro/active-Cathepsin-D levels as sensors of pH decrease, we showed that, in late endosomes, Celecoxib retarded Cathepsin-D activation. This retard was paired by the accumulation of Rab7 and intact EGFR in the lysosomes-enriched fraction of Celecoxib-treated TAFs. These findings suggested that Celecoxib could affect the maturation of late endosomes/autophagosomes contrasting the lowering of pH in these vesicles and/or in lysosomes. Since the acidification/maturation of endosomes relies not only on the H^+^ influx triggered by V-ATPase, but also on the contemporary associated mobilization of chloride, sodium, potassium and calcium ions, Celecoxib could also exert its activity as a known inhibitor of several cationic channels [72, 73].

Our data show that, in colon carcinoma TAFs, chronic Celecoxib treatment exerts a complex control on EGFR levels, activity and turnover. This modulation determines an amplified EGF binding, internalization and signaling, inducing both short (i.e. adhesion) and long-term (i.e. proliferation) biologically relevant responses in colon TAFs. This information should be taken into consideration for any therapeutic regimen involving chronic Celecoxib administration, especially when the upregulation of EGFR could be detrimental. Yet, the use of Celecoxib should be carefully evaluated in those therapeutic regimens including EGFR inhibitors. In our previous work we showed that the EGFR tyrosine kinase (tyr-k) inhibitor Tyrphostin blocked the EGF+Celecoxib synergy more efficiently than Cetuximab [32]. As Cetuximab activity is mainly mediated by EGFR internalization and degradation [31], Celecoxib could lower the efficiency of this process, while tyr-k inhibition would be unaffected. Another way Celecoxib could alter Cetuximab efficacy is through the increased expression of EGFR on colon TAFs: *in vivo*, tumor stroma wrapping epithelial cancer cells could sequester and lower the levels of drug available for cancer targeting. Last but not least, Celecoxib prescription for pain relief in arthrosis could condition EGFR levels in the intestine of elderly patients already affected by different gut pathologies, possibly influencing the course of the disease and therapy. While these hypotheses need further investigations, the “Celecoxib lesson” indicates the need for a detailed identification of the off-target effects of new COX-2 inhibitors based on a similar chemical scaffold.

## MATERIALS AND METHODS

### TAFs and CRC cell lines

MF1T, MF2T and MF3T primary human colon TAFs cell cultures from colon adenocarcinomas were previously described [32]. Most of the assays showed in this study were performed on MF2T, and were confirmed with MF1T and MF3T TAFs. MF2N (MAF), derived from normal colon mucosa, were used as an additional control in Fig. 1S. The colorectal cancer (CRC) cell lines CaCo2 and HT29 (COX-2 positive), HCT15 and SW480 (COX-2 negative) were cultured in DMEM 10% FCS. CaCo2 were also differentiated by long-term confluent *in vitro* culture [74]. CRC cell lines, except for differentiated CaCo2, were tested at low confluence, in the same conditions described for TAFs.

TAFs, at passage 4 of *in vitro* culture, were plated in DMEM 10% FCS and let to adhere for 24h-48h. Before any assay, except for growth assays, TAFs were serum-starved in RPMI with 25mM Hepes buffer (SFM = serum free medium) for 24h to reduce basal signaling. After starvation, SFM was changed and TAFs were treated with Celecoxib (Alexis, 10 μM) for 24 - 48h. TAFs were treated with EGF (Peprotech, 50ng/ml) for the indicated times.

### Cell growth assay

The assay was run as described previously [32]. Briefly, TAFs (4,000 cells/well) were plated in 96 well plates (Nunc) in RPMI 25mM HEPES containing 1% FCS. The presence of FCS was necessary to avoid TAFs layers contraction and detachment along the 7 days culture of the assay. Two hours after plating, TAFs were treated with Celecoxib, EGF or both. Growth assay was stopped using crystal violet fixing-staining solution (4% paraformaldehyde, 30% ETOH, 60mM NaCl, 5g/l crystal violet in H_2_O). Staining was eluted (50% ETOH, 0.1% CH_3_COOH in H_2_O) and quantified by a spectrophotometer at 595nm (VersaMax, Molecular Devices).

### Cell adhesion assay

TAFs adhesion was assessed in 96-wells plates (Nunc), not treated for cell culture and coated with type IV collagen (Sigma, 5μg/ml in H_2_O 0.1% CH_3_COOH). TAFs were primed or not with Celecoxib in SFM for 24h in the tissue culture flask and plated in the adhesion plate with or without EGF (10,000 cells/well, four replicates for experimental point) for 30min. At the end of the incubation TAFs were washed with PBS and fixed/stained with crystal violet fixing-staining solution. Eluted staining was quantified by spectrophotometry (595nm).

### Real time PCR

Total RNAs were obtained from colon TAFs pretreated or not with Celecoxib for 48h with/without EGF (50ng/ml, added 16h before stopping the test). RNAs were extracted and reverse transcribed with oligo(dT) primers as described [32]; EGFR mRNA expression was analyzed by quantitative real-time reverse transcription-PCR by using the following primers: sense 5′- ACTGCTGCCACAACCAGTG and antisense 5′-GGCTTCGTCTCGGAATTTG. The relative expression of EGFR was assessed in comparison with the housekeeping gene RNAP2 (RNA polymerase 2) amplified with the following primers: sense 5′-GACAATGCAGAGAAGCTGG and antisense 5′-GCAGGAAGACATCATCATCC. cDNAs amplification and relative expression values were obtained as described [75].

### Western blot

Cell lysates of colon TAFs, pretreated or not with Celecoxib (or MG132, Bafilomycin-A1, NH_4_Cl) for 48h with/without EGF (50ng/ml, added 16h before stopping the test), were obtained in RIPA buffer, resolved (15-20 μg/lane) on 10% SDS PAGE precast gels (Thermo Scientific) and blotted on PVDF membranes (GE-healthcare). Primary antibodies: anti p-Akt (ser473), p-Erk1-2 (thr202/tyr204), EGFR (D38B1), IkBα were from Cell Signaling Technology; anti EGFR (sc-03), p62/SQSTM1 (D3), HSP70 (3A3), Rab7 (H50) were from Santa Cruz Biotechnology; anti EEA1 (14-EEA1, BD Biosciences); anti LAMP1 (H4A3, Developmental Studies Hybridoma Bank, University of Iowa, Iowa City, IA); anti Cathepsin-D (Calbiochem). HRP-conjugated secondary antibodies (Cell Signaling) were used according to the manufacturer instructions and protein bands were detected by chemiluminescent HRP substrate (Immobilon Western, Millipore) and Hyper film-ECL (GE-healthcare). Anti beta-actin (HRP-conjugated, Cell Signaling Technology) was used as loading control. MG132 and Bafilomycin-A1 were from Sigma. Densitometric quantification of bands was obtained by Image-J (http://imagej.nih.gov/ij/download.html). For statistical analysis and graphical representation of multiple western blot (WB) experiments, each data set was normalized against the sum of all data points in a replicate according to Degasperi et al. [76]. In Fig. 2c and 4 c, 4e untreated controls were set to 1, to get an immediate representation of fold variations of the other data. Results were plotted as mean+/− s.e.

### Flow cytometry

Colon TAFs were pretreated in the same conditions described for Real Time PCR and western blot (Fig. 2), or immunofluorescence (Fig. 3). At the end of incubation, TAFs were harvested with trypsin, pelleted and immediately resuspended/fixed in PBS 1% PAF (10 min, 4°C). For intracytoplasmic EGFR evaluation an aliquot of each cell sample was permeabilized with Triton-X 100 (0.1% final concentration, 10 min, 4°C). Surface EGFR was detected using Cetuximab (2 μg/ml) as primary antibody followed by a goat anti-human immunoglobulin antiserum conjugated with AlexaFluor-647 fluorochrome (Molecular Probes, Life Technologies) as secondary antibody. Total EGFR in permeabilized TAFs was detected by direct staining with anti EGFR-AlexaFluor-647 rabbit mAb (Cell Signaling), targeting an intracellular domain of the receptor not influenced by EGF binding. The intracellular staining with Cetuximab was also run in parallel as an additional control. Samples were run on a CyAn ADP flow cytometer (Beckman Coulter) and at least 5000 events for each sample were analyzed with the Summit v4.3software. A relative quantification of EGFR expression was obtained calculating the ratio of MFI between each Celecoxib and/or EGF treated sample, and the untreated control. Negative controls were always below the first Log of expression and were not plotted in the figures.

### Binding/internalization

EGF binding and internalization assays were run in parallel and measured by a non-radioactive method [77], based on biotin-EGF (Invitrogen). Colon TAFs were plated in two 96-wells plates (20000/well); after 48h cells were switched to SFM for 24h and successively incubated for additional 24h in the presence/absence of Celecoxib. At the end of incubation TAFs were washed twice with cold PBS (with Ca^++^ and Mg^++^) and incubated with 50ng/ml biotin-EGF in SFM (4°C, 45min for binding and 37°C, 30min for internalization). The plate used for the internalization test was then incubated in acidic buffer, pH 3, to eliminate residual membrane-bound EGF. After two consecutive washings with cold PBS, TAFs were fixed and permeabilized. Residual binding sites were blocked by two consecutive incubations with glycine 50mM in PBS and Gelatin 2% + Tween20 0.05% in PBS. Biotin EGF was revealed by incubation with streptavidin-HRP (Life Technologies) 1:15.000 dilution. After extensive washings, TAFs were incubated with the Substrate Reagent Pack DY999 (R&D) and then blocked with DY994 stop solution. Gemini VersaMax spectrophotometer was used to quantify the staining at 450nm. Each experimental point was run in six replicates and data were normalized against controls processed in parallel, either in the absence of TAFs, or with TAFs without biotin-EGF incubation.

### Immunofluorescence and image analysis

Colon TAFs were plated on 20mm diameter glass coverslips (100000/35mm petri dish, in 3ml DMEM 10% FCS). After 3 days TAFs were switched to SFM, 24h later they were treated or not with Celecoxib for 48h. EGF was then added to TAFs and cells were incubated for 30, 90, 180min or 16h at 37°C. TAFs were fixed in PAF 4% for 20min at room temperature and blocked/permeabilized in PBS 5%FCS, 1%BSA, 0.3%Triton-X 100. TAFs were incubated with anti EGFR (rabbit polyclonal, Santa Cruz Biotechnology, 1:500) and anti EEA1 (mouse IgG1, BD, 1:5000) antibodies in PBS 1%BSA, 0.3%Triton-X 100, 4°C overnight. Anti-mouse IgG1 AlexaFluor-488 (1μg/ml) and anti-rabbit AlexaFluor-594 (2 μg/ml) were used as secondary antibodies. Nuclei were counterstained with DAPI. Fluorescent images were acquired, with fixed exposition, by a Leica DM-LB2 microscope equipped with I3 and M2 filters, HCX PL Fluotar 20X, 40x and 100x (for oil immersion) objectives and an Olympus DP70 digital color camera.

Six random fields (taken with the 40x optic) per condition were analyzed by Image-J, to calculate M1 and M2 Mander's overlay coefficients. Briefly, the red (for AlexaFluor-594 = EGFR) or green (for AlexaFluor-488 = EEA1) channels of paired RGB images were extracted and the relative threshold was calculated by the *RenyiEntropy* algorithm. This restrictive algorithm was selected to allow the analysis of medium-large endosomes, eliminating most small-diameter particles (early endosomes) where the overlay of EEA1 and EGFR was almost absolute. M1 an M2 Mander's coefficients were calculated using the JACoP plug-in (http://rsb.info.nih.gov/ij/plugins/track/JACoP_.class). Coefficients were plotted to describe the time-dependent and reciprocal variations of co-localization between EGFR and EEA1.

### Subcellular fractionation

Crude lysosomal and endosomal fractions were isolated by differential centrifugation following published procedures [78, 79], with slight modifications. Briefly, TAFs (10×10^6^) were washed with ice-cold PBS and scraped in 5ml PBS. The cell pellet was resuspended in homogenization buffer (250mM sucrose, 0.5 mM ethylene glycol-bis(β-aminoethyl ether)-N,N,N',N'-tetra acetic acid (EGTA), 20mM Hepes-KOH pH 7) and passed sequentially through a 21G1/2 needle (20 strokes) and a 25G needle (20 strokes) fitted to a 1ml syringe. The homogenate was centrifuged 10min at 1,000xg and 2min at 8,000xg. The post-nuclear supernatant was further fractionated by ultracentrifugation in a TL-100 ultracentrifuge equipped with a TLA-100.3 fixed angle rotor: 2min at 37,000xg (lysosome-enriched fraction), 6min at 50,000xg (late endosome-enriched fraction), 90min at 100,000xg (early endosome-enriched fraction). Fractions were resuspended in RIPA buffer with protease inhibitors and immediately processed for western blot analysis. Protein content was assessed by the DC protein Assay (BioRad) and 10 μg of proteins were loaded on 10% Tris-glycine gels. Each enriched fraction was verified by the relative levels of EEA1, Rab7, LAMP1 and Cathepsin-D, while rough post nuclear supernatant lysate (Pns) was used as loading/staining control to normalize and plot data.

### Statistics

Data were analyzed by two-tailed *t-test* and standard *p* (*p ≤ 0.05; **p ≤ 0.01; ***p ≤ 0.001) are shown on graphs.

## SUPPLEMENTARY MATERIAL AND FIGURES


