# Supplementary material for: Celecoxib increases EGF signaling in colon tumor associated fibroblasts, modulating EGFR expression and degradation
Source: Oncotarget. 2015 Mar 29;6(14):12310–25. doi: 10.18632/oncotarget.3678 (PMC4494940; doi:10.18632/oncotarget.3678)
Supplement: Supplementary file 1 [file oncotarget-06-12310-s001.pdf]

Celecoxib increases EGF signaling in colon tumor associated fibroblasts, modulating EGFR expression and degradation

Supplementary Material

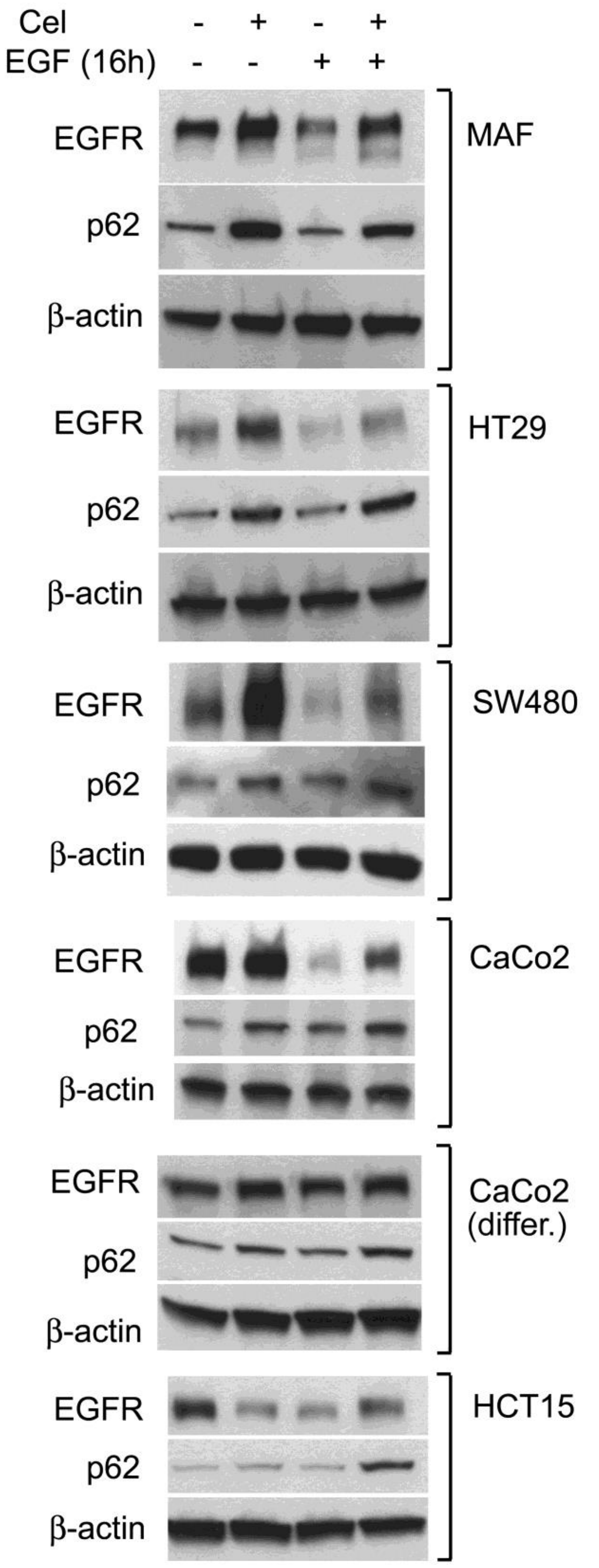

Figure S1: Modulation of EGFR by Celecoxib in normal colon mucosa and CRC cell lines

Normal colon mucosa associated fibroblasts (MAF) and four CRC cell lines were incubated with Celecoxib for 48h; 16h before incubation stop EGF (50ng/ml) was added as indicated. Total cell lysates were tested in western blot for EGFR and p62 expression. CaCo2 cells differentiated in long-term confluent culture were also tested.

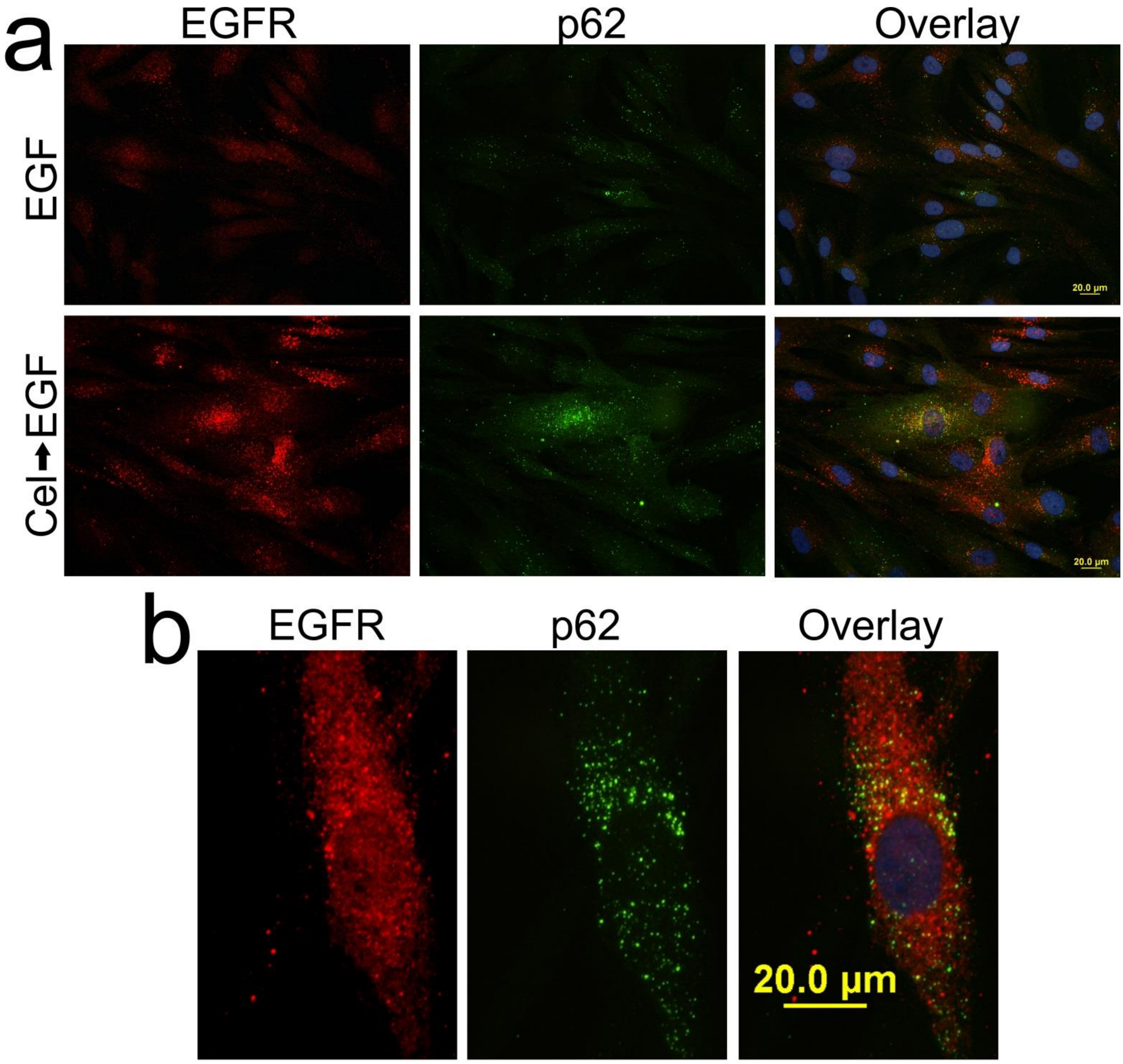

Figure S2: Double immunofluorescence for EGFR (red) and p62 (green)
TAFs were pretreated with Celecoxib 10µM and triggered with EGF (50ng/ml) for 30, 90 and 180 min. Fluorescent images were acquired, with fixed expositions by a Leica DM-LB2 microscope equipped with I3 and M2 filters and a HCX PL Fluotar 40x non immersion optic.
a) Representative images (90 min EGF incubation) of the double immunofluorescence are shown. Autophagy is poorly activated in colon TAFs as p62 forms few autophagosomes per cell. Rare cells with intense p62 clustered staining were observed.
b) Magnification of a TAF (analysed as in a) showing active autophagy: the overlay between p62 and EGFR is infrequent and most EGFR is clustered in p62 negative vesicles.
